# On Tumors of the Maxilla, and Amputation of This Organ by the Subcutaneous Method

**Published:** 1869-02

**Authors:** Charles Menecier


AMERICAN JOURNAL

OF

DENTAL SCIENCE.

Vol. 11. THIRD SERIES—FEBRUARY, 1869. No. 10.

### ARTICLE I.

### *On Tumors of the Maxilla, and Amputation of this Organ by the Subcutaneous Method.*

By Dr. Charles Menecier.

(Translated from the French for the American Journal of Dental Science.)

Surgical Pathology may lay claim to a great proportion of the diseases which attack the lower jaw. From simple or gunshot fractures, to organic degeneration, the range is great, but all these affections have not the same importance for the patient nor the same interest for the surgeon.

I do not intend, in this article, to treat of all the diseases of this bone; it suffices to cite those commonly met with to show the impossibility of such an attempt. Thus, in one of the last numbers of the *Sud Medical*, our colleague, Dr. Olive, has brought forward a new apparatus which, in a very complicated case, has perfectly succeeded, in his hands, in consolidating complicated fractures of the lower jaw at its middle portion. This is a point of conservative surgery always desirable to attain, but unfortunately we often meet with such grave disorders, that we are obliged to resort to the most difficult operations to save the patient from imminent death.

The numerous tumors of the lower jaw may give some idea of the frequency of practicable operations on this portion of the face. The rarest of these tumors are unquestionably the lipomas. Nelaton discovered a remarkable case in the dead body. M.M. Ch. Robin and Follier say they have only extirpated two of them in the living patient. Next come the cysts, the science of which is not in a very stable condition. Those who have specially studied them have put forth nothing but hypotheses concerning them. Osteoma is very frequent, but, like fibroma, its development is so slow that very often it is unnecessary to interfere. In these two classes of tumors, the salient subjective symptom is the violent pain which frequently accompanies them, a pain produced in osteoma by the contraction of the dental canal, and in inter-osseous fibroma, by the direct compression of the nerve in this same canal.

Immediately after these come the medullary tumors, which usually originate about the alveoli, either under the periosteum or in the proper tissues of the bone. Under this head, we classify enchondroma (osteo-chondrophyte of Cruveilher) long confounded under the title of *spina ventosa*, and plasmoma or fibro-plastic tumor, the tendency of which towards ulceration is sufficiently common. Besides the prognosis of the these two groups of tumors differs essentially from the first in their tendency to undergo suddenly a rapid evolution which may require the intervention of the surgeon. In this respect they approach malignant tumors, like cancer, the rapid progress of which can sometimes be arrested only by a bloody operation, without which the patient hastens to certain death. We have now among our patients an example of this rapid progress of an epithelioma (smokers' cancer) of the lower lip, which has reached the lower jaw through the mental foramen.

The lower jaw may also be the seat of caries, a necrosis which unfortunately demands an operation, the gravity of which depends entirely upon the extent of the disease. Upon these necroses I propose to dwell somewhat at length, especially upon those produced by the vapors of phosphorus.

The manufacture of matches, an important branch of industry at Marseilles, should be ranked among the most unwholesome of occupations, not so much in reference to its effect upon the public health, as upon that of the work people engaged in it. The numerous girls employed in these factories are not all equally exposed to the pernicious emanations of phosphorus. Age, temperament, anæmia, constitutional debility, prolonged exposure to fumes, all have their influence in different degrees, in aggravating the symptoms of these poor creatures, already weakened by wretchedness and over-work.

Among the young men, poisoning by phosphorus is often first recognized by pains in the testicles, sometimes simulating an orchitis which rest and soothing applications speedily relieve. I have seen the symptoms well marked in a youth of 20 or 22 years, of lymphatic temperament employed in dipping the matches in one of the shops. Women exposed to the same fumes in packing the matches in boxes, experience trouble in their menstruation. This is at first simply more abundant; then the interval between the periods first diminishes and then disappears, so that these girls have a constant dribbling, with intermittent hæmorrhages, which bleach them and rapidly reduce them to extreme anæmia.

It is plain that in persons liable to these disorders, the irritating action of vapors of phosphorus may occasion very grave disturbances. The peri-alveolar periosteum first inflames, detaches the gums, denudes the bone. When the disease attacks the lower jaw, its course is always more rapid than in the other bones of the face. In the early stages, the patient is concious of no symptoms; it is only when some progress has been made that toothache reveals the existence of necrosis of which the work-woman is hard to convince. Unfortunately the extraction of a molar tooth which troubles her brings on the acute stage. The alveolar border is more or less compromised during the operation, the gum does not cicatrize, it even detaches itself rapidly in consequence of the periosteal inflammation, as I have already said; gradually the separation increases, the teeth grow loose, fall out, and leave the alveoli uncovered.

The history of the disease is not always so melancholy. In the better class of patients, vigorous, or at least less debilitated, the necrosis may be self-limited and terminate by elimination of the sequestrum. I believe that this favorable result would be oftener witnessed if the patients would permit, at the commencement, the removal of the necrosed portion. In this manner, I have been able to save for a young man his lower jaw, already much diseased, by removing sequestra from among the alveoli of the two last molars. Unfortunately, this is not always the case. Usually, the patient refuses to be operated upon until the necrosis has made serious inroads. Sometimes even death carries off these unhappy laborers before they can bring themselves to submit to an operation.

In the following case a temporizing policy would have cost the patient her life.

On the 1st of June, 1867, Miss E. M., aged twenty-three years, presented herself at the Central Dispensary with a necrosis of the left lower jaw, produced by phosphoric vapors. She told me she had worked thirteen years in match factories. She had a nervous and lymphatic temperament and commenced menstruating at fourteen. Her constitution is delicate, but none of her family had ever had any serious disease, and she herself had never been sick. For several months, menstruation has been more abundant and her strength has considerably declined, but she always kept on working, labor being her only resource. During the Spring of 1868, she suffered much from the evolution of the wisdom tooth on the left side. The pain became continuous and inflammation of the cheek set in. About the month of August, of the same year, she consulted a dentist, who forthwith extracted the tooth.

After this operation, the pain did not completely disappear, it spread over the whole left side of the lower jaw, which swelled from behind forward, and from below upwards, reaching the ascending ramus. The alveolus never closed but became the seat of an abscess. The pus undermined the bone and finally pointed behind the angle of the jaw upon the neck. At the same time, all the teeth of that side loosened and fell out without being diseased. The pus detached the gums and gathered under the chin. The jaw became more and more immovable, so that the patient could not open her mouth. Nutrition in consequence was much impaired.

Such was the condition of Miss M. on the 1st of June, 1867, when she consulted me. Her general condition was no better. In her chest were some unfavorable stethoscopic sounds, her complexion was earthy, her pulse thready, her strength gone, her appetite lost; finally the menstrual dribbling had been continuous for several months.

In spite of the urgency of the patient who demanded an operation, I was compelled to refuse it. I merely made two openings with a lancet below the jaw, to give exit by natural drainage to the fetid pus which the patient was continally swallowing. I put her upon iron, (iron by hydrogen), bark, roast meat, and frequent washing with decoction of cochlearia and flowers of arnica. After four months of this treatment, I called in consultation my colleagues of the Dispensary, together with my friend Dr. F. Bontonx. The menorrhagia had disappeared. The patient's appetite was good, the pus was laudable, the necrosis limited in front by the symphysis of the chin. It was decided, in view of the notable improvement consequent upon the treatment, to postpone the operation in hopes of still further amendment.

On the sixteenth of November another consultation was held, and it was decided to operate as soon as practicable. The girl had not menstruated, but she could take long walks without fatigue and attend to her household duties. The mucous membrane of the mouth recovered a lively tint; the pus was very laudable, though always excessively fetid. I insisted the more upon the operation because I had thought I

saw a tendency on the part of the necrosis to cross the symphysis of the chin, to attack the right side of the lower jaw, and to extend backward to the zygomatic process or the upper jaw of the left side.

In spite of the great improvement in the patient's general health, I always dreaded a hæmorrhage from the arteries during the operation which might bring about a fatal syncope. To avoid this terrible accident I ordered syrup of perchloride of iron, which she took till the day fixed for the operation, viz : the 5th of December.

I adopted, with modifications, the subcutaneous method for disarticulating the jaw. This plan, proposed in 1847 by Professor Gorgone, in a case of resection of this bone, has the double advantage of but slightly mutilating the face and of avoiding vessels which, when opened, weaken the patient, while prolonging the operation by the ligatures required.

The operation was performed in the presence of my colleagues. The girl was sitting, her head supportod behind. The closing of the jaws and the anæmia forbade the use of chloroform. I made an incision commencing half an inch behind the facial artery, at the point where it turns round the ramus of the lower jaw. This incision directed at first horizontally towards the angle of the jaw, was carried vertically from this point to a third of an inch in front of the *meatus auditorius externus.* At first the skin only was cut. I thought it wrong to dissect off the flap, without first detaching the inferior insertion of the masseter. At this moment the masseteric artery threw out a jet of blood, which I instantly stopped by compression. At the upper angle of the incision, I partly divided the parotid gland to reach the articulation. The triangular flap was detached, not with the bistoury, but with the nails and raspatorium. I effected the section of the attachments of the internal pterygoid muscles with a strong, blunt, pointed bistoury bent like a pruning knife, which had been expressly constructed for the purpose. This same knife answered perfecly for the dissection of the tissues in the neighborhood of the condyle, the most difficult part about this operation, because of the presence of the external carotid and the internal maxillary arteries. The inferior dental artery and nerve were both atrophied.

Passing to the anterior region, I forcibly drew down the lip, after having detached the gum, and I passed the chain saw to cut the jaw in the lymphysis itself. I returned then to the ramus of the maxilla which had been laid bare. The point was to shape it out by the chain saw. This was difficult, as the tissues of the cheek, very œdematous, opposed by their inelastic character, the separation of the lips of the wound. The bone finally was imbedded in a thick, spongy, newly formed layer of bony tissue, which permitted the passage backward of none of the ordinary instruments. With gouge and mallet I pared off a layer of this tissue, I could then pass Belloc's sound without fear of injuring any artery. The sequestrum though resisting, was soon divided. This double section before and behind allowed the patient to open her mouth and enabled me to enucleate the horizontal branch of the jaw with my curved bistoury and the rasp. Fearing that the tongue would separate from its last attachments to the jaw and suffocate my patient, I passed a silver wire through the frenum linguæ and twisted the two ends strongly round the two right incisor.

There still remained a considerable portion of the ramus of the jaw to disarticulate. There again I had to employ the gouge and mallet to divide the bony shell and allow me to cut the attachments of the temporal muscle and external ligament of the articulation, in order to get sufficient play to separate the condyle from the glenoid cavity with strong pincers.

In spite of the inherent difficulties of this particular case, the operation lasted only sixty-five minntes, and the patient had no syncope. The wound of the cheek was brought together by a few stitches of interrupted suture. Union took place by first intention. The void left by the removal of the jaw was filled for several days with fine sponges, which were renewed morning and evening. At every dressing the mouth was injected with alcoholized decoctions of quinine.

After the operation, the patient kept her bed only eight days. She has been out for some time and visits me weekly, impatient for the time to arrive when she can have an artificial apparatus for mastication inserted.

